# Knowledge, Attitudes, and Practices of Family Medicine Physicians Regarding the Costs of Common Laboratory Investigations in the Chronic Disease Clinic at King Saud Medical City: A Cross-Sectional Study

**DOI:** 10.7759/cureus.81102

**Published:** 2025-03-24

**Authors:** Saad F Alyahya, Bander Alshehry, Abdullah S Alsuwayeh, Hussam S Aloufi

**Affiliations:** 1 Family Medicine, Riyadh First Health Cluster, Riyadh, SAU; 2 Family Medicine, King Saud Medical City, Riyadh, SAU; 3 Family Medicine, Makkah Family Medicine Academy Makkah Healthcare Cluster, Makkah, SAU

**Keywords:** chronic disease, cost, family physicians, kingdom of saudi arabia (ksa), laboratory analysis

## Abstract

Objectives: To assess the awareness of family medicine physicians regarding the cost of common laboratory investigations in chronic diseases. It also aims to evaluate their awareness of how costs could affect their medical decision-making.

Methodology: This was a cross-sectional study conducted among all family medicine physicians working in the King Saud Medical City. Our data were collected using questionnaires distributed to participants by group members. Both a paper-based version and an online version through SurveyMonkey (Momentive, CA, USA) were used.

Results: A total of 95 family physicians were included in this study, and nearly half of them, 48 (50.5%), were males. The majority, 66 (69.5%), 72 (75.8%), 70 (73.3%), and 73 (76.4%), physicians underestimated the cost of complete blood count, coagulation profile, iron studies, and liver profile, respectively. The least correctly estimated tests appear to be hemoglobin A1C and iron studies. Correct cost estimation was the highest in urine analysis (81, 85.3%). Work experience was significantly associated with the awareness about liver panel cost (P=0.040).

Conclusion: The capacity of family physicians to correctly estimate the prices of various laboratory tests varies significantly, as this study shows. While most doctors predicted the cost of routine procedures, such as urine analysis, accurately, many had trouble with tests like thyroid panels and creatinine clearance. These results highlight the need for doctors to have a better understanding of costs associated with diagnostic tests since this is necessary to support the provision of cost-effective healthcare.

## Introduction

As part of overall hospital budgets, laboratory testing has been on an unrelenting rise for decades. The rising cost of laboratory testing has been a cause of concern for healthcare systems worldwide. In Saudi Arabia, the Ministry of Health's budget for laboratory testing has increased significantly over the last decade [1], leading to a strain on resources. With the implementation of Saudi Arabia's Vision 2030, the need to improve healthcare system outcomes has become even more crucial.

One way to achieve this is by increasing physician awareness of the costs of diagnostic and therapeutic medical care items. By identifying areas where cost education can prevent overutilization, we can improve the long-term quality and effectiveness of the healthcare system. The Health Sector Transformation Program was launched in 2021 under Vision 2030, with a vision for the next five years to restructure the health sector into a comprehensive, effective, and integrated health system that prioritizes the health of individuals and society, including citizens, residents, and visitors [2].

The program is based on the principle of value-based care, aiming to ensure transparency and financial sustainability by promoting public health and preventing diseases. The program's specific objectives include improving access to quality health services by expanding the provision of e-health services and digital solutions, ensuring optimal coverage, and promoting comprehensive and equitable geographical distribution of health services [3,4].

The National Transformation Program (NTP) was designed to boost the economy, and the Vision 2030 development plan covers economic development at all levels, including healthcare. This plan encourages more private partnerships and privatization of government services. The NTP aims to increase the private sector's contribution to the gross domestic product (GDP) from 40% to 65% by 2030. The program's main healthcare objectives are to improve access to healthcare services, enhance the quality and efficiency of healthcare services, and promote disease prevention through better access to care and preventative services [4,5,6].

Over the last 13 years, the Ministry of Health budget increased from around 25 million riyals (one Saudi riyal equals 0.27 United States dollars) in 2008 (5.6% of the total state budget) to more than 79 million riyals in 2021 (8.2% of the total state budget) [1]. With Saudi Arabia's Vision 2030, one of the most important goals is to improve the quality and efficacy of the healthcare system facing the accelerated worldwide economic depression and limited resources, accompanied by overutilization of lab investigations, which will lead to poor healthcare system outcomes. A systematic review of 14 studies found that the physician's awareness of diagnostic and therapeutic medical care cost items could be better. Cost accuracy was low; 33% of estimates were within 20% or 25% of the actual cost, and 50% were within 50% or 50-200% [7].

Previous studies have discussed the physician's awareness of common medical care costs. A questionnaire study that included junior and senior physicians in 99 French intensive care units was asked to estimate the hospital costs of 46 selected prescriptions commonly used in critical care practice. The article aims to evaluate current intensivists' knowledge of the costs of common prescriptions and to identify factors influencing the accuracy of cost estimations. The finding shows that intensive care unit physicians need a better awareness of prescription costs, especially high-cost drugs. Considerable emphasis and effort are still required to integrate the cost containment problem into the daily prescriptions in ICUs [8].

Physicians will be more cautious when ordering tests if they know the cost of each test. Physicians who are more aware of healthcare costs will help decrease costs without harming patients and improve the healthcare system's quality and efficacy in the short and long term. By understanding the current level of physician cost awareness of common lab investigations, we aim to identify areas where cost education would prevent overutilization of lab investigations done by family medicine physicians to improve healthcare system quality and efficacy in the long run. This study aims to assess family medicine physicians' awareness of the cost of common lab investigations in chronic diseases and assess how awareness of costs impacts their medical decision-making.

## Materials and methods

Study design and setting

This study was a cross-sectional study, conducted in Riyadh, Saudi Arabia, at King Saud Medical City. The study was conducted over a period of six months, from January 2024 to June 2024, at King Saud Medical City. Data collection took place between February 2024 and May 2024, while data analysis was completed by July 2024.

Study subjects

Family medicine physicians who work at King Saud Medical City are considered part of our population.

Sample size

Our population is 100 doctors, the confidence level is 95%, the margin of error is 5%, and according to this law:

\[ x = Z\Bigl(\frac{c}{100}\Bigr)^{2} \times r \times (100 - r) \]

\[ n = \frac{N \times x}{(N - 1)\,E^{2} + x} \]

\[ E = \sqrt{\frac{(N - n)\,x}{n \times (N - 1)}} \] Our estimated sample size was 80 doctors. The sampling technique was conducted using consecutive (non-probability) sampling.

Data collection

The data was collected using a chart review. Our source of data was taken as questionnaires given to the population by group members (SA, AA, HA) in informed sheets that can be distributed among family medicine physicians. This was a self-developed questionnaire. The questionnaire was designed based on a literature review and expert consultation. Content validity was ensured by reviewing the items with specialists in family medicine and healthcare economics. A pilot study was conducted with 10 physicians to test reliability, yielding a Cronbach’s alpha of 0.82, indicating good internal consistency. The questionnaire was originally developed in English. The study’s questionnaire was included in the supplementary files, and doctors filled out both a paper-based version and an online version through SurveyMonkey (Momentive, CA, USA) during their clinic hours (Appendix 1, 2).

Ethical considerations

The subjects of this research were fully aware of the nature and purpose of the research project. During the study, subjects were informed of the precautions that were taken to protect the confidentiality of the data. Security procedures (e.g., encryption, password protection) were practiced when patient data was transferred into SPSS on a computer. The information that identified the patient will be removed for example: names, medical record numbers (MRN), telephone numbers, etc. A code was opted to replace the identifying information of the individual with a number, letter, symbol, or some combination. This study was voluntary, and volunteers were free to withdraw at any time. This research is IRB-approved with IRB Registration Number H1RI-06-Nov23-02.

Statistical analysis

IBM SPSS Statistics for Windows, Version 26 (Released 2019; IBM Corp., Armonk, New York, United States) was used to code, enter, and analyze all of the data, computed frequencies and percentages for categorical data, like "gender." Correlations were evaluated using the chi-square test. If a test's p-value was less than 0.05, it was considered significant.

## Results

A total of 95 family physicians were included in this study, and nearly 48 physicians (50.5%) were males. Almost 36 physicians (37.9%) were in the first year of residency, 77 physicians (81.1%) never worked in private hospitals, 49 physicians (51.6%) had less than two years of experience, and 49 physicians (51.6%) agreed that common knowledge about the financial cost of common lab investigations can affect part of the clinical practice (Table 1).

**Table 1:** Sociodemographic characteristics of the included physicians (n=95). Demographic and professional characteristics of the 95 participating physicians. Data are presented as frequency (N), and percentage (%). These data are presented as categorical variables (e.g., gender, current position, prior private‐hospital experience). The standard test statistic to compare distributions or evaluate associations between such categorical variables is the chi-square test. A p-value <0.05 was considered statistically significant, with p<0.001 denoting a highly significant result. R1: first-year residency; R2: second-year residency; R3: third-year residency.

Parameter		Frequency (%)
Gender	Male	n=48 (50.5%)
	Female	n=47 (49.5%)
Current position	R1	n=36 (37.9%)
	R2	n=24 (25.3%)
	R3	n=18 (18.9%)
	Specialist	n=9 (9.5%)
	Consultant	n=8 (8.4%)
Have you ever worked in private hospitals?	Yes	n=18 (18.9%)
	No	n=77 (81.1%)
Work experience	<2 years	n=49 (51.6%)
	2-5 years	n=31 (32.6%)
	5-8 years	n=6 (6.3%)
	>8 years	n=9 (9.5%)
Knowledge about the financial cost of common lab investigations can affect part of the clinical practice	Strongly agree	n=29 (30.5%)
	Agree	n=49 (51.6%)
	Disagree	n=4 (4.2%)
	Strongly disagree	n=3 (3.2%)
	Neither agree nor disagree	n=10 (10.5%)

**Figure 1:**
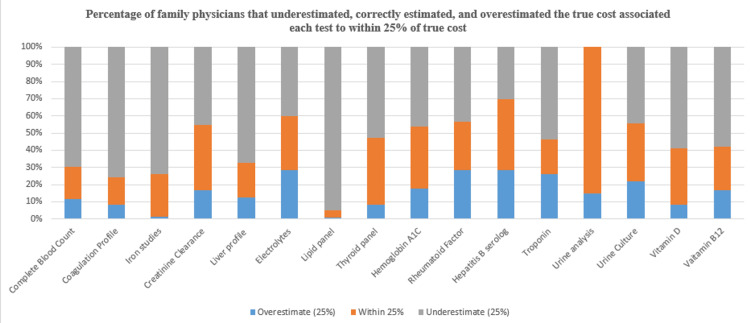
The percentage of family physicians who either overestimated, correctly estimated (within 25% accuracy), or underestimated the true costs of various laboratory tests.

For most tests (such as complete blood count, coagulation profile, iron studies, and creatinine clearance), the majority of physicians underestimated the costs, as indicated by the large gray sections in the bars. Certain tests, like urine culture and urine analysis, show a higher proportion of correct estimation (orange), suggesting better cost awareness among physicians for these tests. Overestimation (blue) of costs is more common for specific tests like vitamin B12, vitamin D, and troponin, indicating that some physicians tend to believe these tests are more expensive than they are. The least correctly estimated tests (with smaller orange portions) appear to be hemoglobin A1C and iron studies, highlighting a greater discrepancy in cost awareness for these particular tests (Figure 1).

Around 66 (69.5%), 72 (75.8%), 70 (73.3%), and 73 (76.4%) physicians underestimated the cost of CBC, coagulation profile, iron studies, and liver profile, respectively. Correct cost estimation was the highest in urine analysis (81, 85.3%), followed by thyroid panel (37, 38.9%) and creatinine clearance (36, 37.9%). Hence, the least estimation error was 14 (14.7%) in the urine analysis investigation (Table 2).

**Table 2:** Cost estimation by test. Number (N) and percentage (%) of physicians who overestimated (≥25% above actual cost), correctly estimated (within ±25% of actual cost), or underestimated (≥25% below actual cost) the true cost of each laboratory test. The “Median % error” column reflects the median deviation of the estimated cost from the actual cost. A chi-square test is used to compare the distribution of responses (overestimate, correct, underestimate) across multiple laboratory tests. This determines if physicians’ estimations differ significantly among tests beyond what would be expected by chance. p < 0.05 is considered statistically significant, and p < 0.001 is considered highly significant. (*) → Statistically significant difference in cost estimation. (**) → Highly significant difference in cost estimation.

Test name	Overestimate (25%) (N, %)	Within (25%) (N, %)	Underestimate (25%) (N, %)	Median % error	Chi-square (χ²) Value	p-value
Complete blood count	11 (11.6%)	18 (18.9%)	66 (69.5%)	18.9	χ² = 15.32	0.002**
Coagulation profile	8 (8.4%)	15 (15.8%)	72 (75.8%)	15.8	χ² = 13.75	0.005**
Iron studies	1 (1.1%)	24 (25.3%)	70 (73.7%)	25.3	χ² = 20.21	0.001**
Creatinine clearance	16 (16.8%)	36 (37.9%)	43 (45.3%)	37.9	χ² = 9.87	0.045*
Liver profile	12 (12.6%)	19 (20.0%)	64 (67.4%)	20.0	χ² = 14.56	0.003**
Electrolytes	27 (28.4%)	30 (31.6%)	38 (40.0%)	31.6	χ² = 7.92	0.048*
Lipid panel	6 (6.3%)	26 (27.4%)	63 (66.3%)	27.4	χ² = 11.65	0.022*
Thyroid panel	8 (8.4%)	37 (38.9%)	50 (52.6%)	38.9	χ² = 8.49	0.034*
Hemoglobin A1C	17 (17.9%)	34 (35.8%)	44 (46.3%)	35.8	χ² = 10.72	0.029*
Rheumatoid factor	27 (28.4%)	27 (28.4%)	41 (43.2%)	28.4	χ² = 6.73	0.068
Hepatitis B serology	27 (28.4%)	39 (41.1%)	29 (30.5%)	30.5	χ² = 5.92	0.072
Troponin	25 (26.3%)	19 (20.0%)	51 (53.7%)	26.3	χ² = 9.42	0.041*
Urine analysis	14 (14.7%)	81 (85.3%)	0 (0.0%)	14.7	χ² = 27.63	<0.001**
Urine culture	21 (22.1%)	32 (33.7%)	42 (44.2%)	33.7	χ² = 7.35	0.056
Vitamin D	8 (8.4%)	31 (32.6%)	56 (58.9%)	32.6	χ² = 10.83	0.027*
Vitamin B12	16 (16.8%)	24 (25.3%)	55 (57.9%)	25.3	χ² = 11.12	0.024

## Discussion

The results from our study suggest that Saudi family physicians felt that they did not have the appropriate understanding regarding diagnostic and laboratory test prices, and, in fact, most physicians could not estimate these costs; for most tests (such as complete blood count, coagulation profile, iron studies, and creatinine clearance), the majority of physicians underestimated the costs, as indicated by the large gray sections in the bars. Overestimation (blue) of costs is more common for specific tests like vitamin B12, vitamin D, and troponin, indicating that some physicians tend to believe these tests are more expensive than they are. The least correctly estimated tests (with smaller orange portions) appear to be hemoglobin A1C and iron studies, highlighting a greater discrepancy in cost awareness for these particular tests. This was in line with Sa et al., who reported that Portuguese family doctors are not well-informed about the expenses associated with laboratory and diagnostic testing [9]. However, these findings are in contrast to those from other nations, where it has been noted that there is a tendency to overestimate the less expensive tests and underestimate the more expensive tests [10,7,11]. The discrepancy does not have a single definitive cause; however, one of the most significant contributing factors is that healthcare services in Saudi Arabia are provided free of charge. As a result, many physicians are not fully aware of the costs associated with most laboratory tests.

We found that correct cost estimation was the highest in urine analysis (81, 85.3%), followed by thyroid panel (37, 38.9%) and creatinine clearance (36, 37.9%). Hence, the least estimation error was 14 (14.7%) in the urine analysis investigation. The high correct estimation for urine analysis likely stems from its frequent use in clinical practice. Tests that are regularly ordered tend to have more standardized pricing, and physicians are generally more aware of their costs. In contrast, tests like thyroid panels and creatinine clearance may not be ordered as routinely by general practitioners, resulting in lower cost estimation accuracy [12].

Work experience was significantly associated with the awareness about liver panel cost (P=0.040), as those who have worked in a private hospital did not report any accurate costs. A significant 82 (86.2%) physicians who accurately estimated costs strongly agreed that cost knowledge impacts clinical practice. Physicians who underestimated costs were more likely to disagree (24, 25%) or strongly disagree (32, 33.3%) with the statement. A neutral stance was more common among overestimators (38, 40%) and underestimators (48, 50%), indicating uncertainty about the influence of cost knowledge on clinical decisions (P=0.022). This was consistent with Allan et al. [7]. The following factors did not affect cost awareness: sex, practice location, faculty appointment, Certification of the College of Family Physicians of Canada (CCFP), practice duration, or answers to the other four statements [7].

Limitations

The study has a small sample size, which could affect the generalizability of the results. A larger and more diverse sample of physicians from various specialties and regions could provide more representative insights. Cost estimations may vary significantly based on geographical location. The study only examined a specific set of laboratory tests, which may limit its applicability to other diagnostic procedures or tests not included in the analysis. The study primarily focused on family physicians, and cost awareness may differ across medical specialties. Including specialists who regularly order specific tests could offer a more comprehensive understanding of cost estimation across disciplines.

Recommendations

The study recommends implementing specialized educational programs and workshops to enhance physicians’ understanding of diagnostic test costs. These initiatives should extend beyond frequently ordered tests to include less common ones, such as thyroid panels and creatinine clearance. By integrating real-time pricing tools into clinical practice, healthcare organizations can improve cost transparency, enabling physicians to make more informed decisions. Introducing cost-awareness training into medical school curricula will further ensure that future doctors recognize the financial impact of clinical choices. Involving multidisciplinary experts in related research efforts would provide a more comprehensive view of cost estimation across various fields.

Although physicians are generally familiar with the costs of standard tests like urine analyses, the findings point to a gap in awareness regarding other diagnostics. Addressing this knowledge deficit through targeted educational efforts could reduce unnecessary testing, encourage cost-effective decision-making, and promote more accurate estimates of test prices. In turn, this heightened awareness could help prevent both the overuse of expensive tests and the underuse of critical diagnostics, ultimately fostering better resource utilization and service quality.

Program leaders at the medical school, residency, and postdoctoral levels should take note of these findings. Various measures, such as educational programs, clinical recommendations, computer-based ordering, evaluation and audits, physician inducement, fundholding, or formulary limits, could be employed in conjunction with cost-awareness initiatives. Forced limits appear to save the most money, but these tactics may have a detrimental impact on health outcomes and increase the system's long-term expenditures.

## Conclusions

The capacity of family physicians to correctly estimate the prices of various laboratory tests varies significantly, as this study shows. While most doctors predicted the cost of routine procedures, such as urine analysis, accurately, many had trouble with tests like thyroid panels and creatinine clearance. These results highlight the need for doctors to have a better understanding of costs associated with diagnostic tests since this is necessary to support the provision of cost-effective healthcare. Closing the gaps through systemic interventions and education may assist in maximizing the use of available resources, reducing the number of pointless tests, and improving the general standard of patient care.

## Appendices

Appendix 1

**Table 3:** Participant form.

You are being invited to participate in a research study titled “Evaluation of family medicine physicians' awareness of the cost of common lab investigations in chronic diseases clinics at King Saud Medical City”. This study is being done by Dr. Bander Alshehry from King Saud Medical City.																													
· Why are we doing this research study?																													
§ The purpose of this research study is to assess family medicine physicians' awareness of the cost of common lab investigations in chronic diseases and assess how awareness of costs impacts their medical decision-making.																													
· Who can participate in this research study?																													
§ Any family medicine staff or general practitioner who is working in King Saud Medical City is eligible for this study.																													
· What will I be asked to do and how much time will it take?																													
§ If you agree to take part in this study, you will be asked to complete an online survey/questionnaire. This survey/questionnaire will ask about the current position, to estimate the costs of common lab investigations, and it will take you approximately five minutes to complete.																													
· Will being in this research study help me in any way?																													
§ Participants in this research will have their own value added to improve our health care and sustainability.																													
· What are my risks of being in this research study?																													
§ No potential risk is expected from such a questionnaire. It will take just five minutes.																													
· How will my personal information be protected?																													
§ To the best of our ability, your answers in this study will remain confidential.																													
· Will I be given any money or other compensation for being in this research study?																													
§ No money will be paid.																													
· What happens if I say yes, but change my mind later?																													
§ You do not have to be in this study if you do not want to. If you agree to be in the study, but later change your mind, you may drop out at any time. There are no penalties or consequences of any kind if you decide that you do not want to participate.																													
· Who can I talk to if I have questions?																													
§ If you have questions about this project or if you have a research-related problem, you may contact the researcher(s).																													
· Do you agree to participate in this study																													
§ ( ) Yes																													
§ ( ) No																													

Appendix 2

**Table 4:** Questionnaire. PT: prothrombin time; PTT: partial thromboplastin time; INR: international normalized ratio; AST: aspartate aminotransferase; ALT: alanine aminotransferase; TIBC: total iron binding capacity; GGT: gamma-glutamyl transferase; LDL: low-density lipoprotein; HDL: high-density lipoprotein; TSH: thyroid-stimulating hormone; SAR: Saudi riyal; R1: first-year residency; R2: second-year residency; R3: third-year residency

Question	Options
1. What is your gender?	( ) Male
	( ) Female
2. What is your current position?	( ) R1
	( ) R2
	( ) R3
	( ) Specialist
	( ) Consultant
3. Have you ever worked in private hospitals?	( ) Yes
	( ) No
4. How many years have you been working as a family physician (started from the first year of work, the earliest option, either as a trainee in a training program or as a family medicine staff)?	( ) less than 2 years
	( ) 2 years to 5 years
	( ) 5 years to 8 years
	( ) more than 8 years
5. I think, if I were informed about the financial cost of common lab investigations, it would affect part of my clinical practice	( ) Strongly agree
	( ) Agree
	( ) Neither agree nor disagree
	( ) Disagree
	( ) Strongly disagree

6. Estimate the cost of these laboratory investigations in Saudi Riyal							
Investigation	0‑100 SAR	101‑200 SAR	201‑300 SAR	301‑400 SAR	401‑500 SAR	501‑600 SAR	>600 SAR
Complete blood count with differential	( )	( )	( )	( )	( )	( )	( )
Coagulation profile (PT, PTT, INR)	( )	( )	( )	( )	( )	( )	( )
Iron studies (ferritin, transferrin saturation, TIBC)	( )	( )	( )	( )	( )	( )	( )
Creatinine clearance	( )	( )	( )	( )	( )	( )	( )
Liver profile (AST, ALT, GGT, albumin)	( )	( )	( )	( )	( )	( )	( )
Electrolyte panel (CHEM‑7)	( )	( )	( )	( )	( )	( )	( )
Lipid panel (HDL, LDL, total cholesterol, triglyceride)	( )	( )	( )	( )	( )	( )	( )
Thyroid panel (TSH, T3, T4)	( )	( )	( )	( )	( )	( )	( )
Hemoglobin A1C	( )	( )	( )	( )	( )	( )	( )
Rheumatoid factor (RF)	( )	( )	( )	( )	( )	( )	( )
Hepatitis B serology (HBsAg, anti-HBs, anti-HBc, IgM anti-HBc)	( )	( )	( )	( )	( )	( )	( )
Troponin	( )	( )	( )	( )	( )	( )	( )
Urinalysis	( )	( )	( )	( )	( )	( )	( )
Urine culture	( )	( )	( )	( )	( )	( )	( )
Vitamin D	( )	( )	( )	( )	( )	( )	( )
Vitamin B12	( )	( )	( )	( )	( )	( )	( )

7. A 61-year-old male K/C DM, HTN, dyslipidemia. He is currently taking metformin 1000 mg, empagliflozin 10 mg, lisinopril 5 mg, and rosuvastatin 20 mg. He has been brought to your clinic for regular follow-up. The last follow-up was nine months ago. His last labs were unremarkable except for hemoglobin, 11; MCV, 70; HbA1c, 8.1; LDL, 150; and serum creatinine, 90 O/E. The patient is conscious, oriented, and alert. Vitals BP: 132/85; HR: 72; Temp: 37.4; RR: 23; BMI: 29 Local examination was unremarkable. What will you order next for this patient?
( ) Complete blood count with differential
( ) Coagulation profile (PT, PTT, INR)
( ) Iron studies (ferritin, transferrin saturation, TIBC)
( ) Creatinine clearance
( ) Liver profile (AST, ALT, GGT, albumin)
( ) Electrolyte panel (CHEM‑7)
( ) Lipid panel (HDL, LDL, total cholesterol, triglyceride)
( ) Thyroid panel (TSH, T3, T4)
( ) Hemoglobin A1C
( ) Rheumatoid factor (RF)
( ) Hepatitis B serology (HBsAg, anti‑HBs, anti‑HBc, IgM anti‑HBc)
( ) Troponin
( ) Urinalysis
( ) Urine culture
( ) Vitamin D
( ) Vitamin B12
( ) No need for laboratory investigations for this patient

8- A 22-year-old woman with a history of asthma presents with a sudden onset of bilateral blindness, denying any trauma. Her mother states, “I can’t believe this is happening. First, her father is dying of cancer, and now this.” On observation, she skillfully avoids obstacles despite her reported blindness. Examination reveals normal bilateral pupillary responses, and there are no signs of bruises or scrapes. A visual evoked potential test was ordered and returned normal. Which laboratory investigations would you order next for this patient?
( ) Complete blood count with differential
( ) Coagulation profile (PT, PTT, INR)
( ) Iron studies (ferritin, transferrin saturation, TIBC)
( ) Creatinine clearance
( ) Liver profile (AST, ALT, GGT, albumin)
( ) Electrolyte panel (CHEM‑7)
( ) Lipid panel (HDL, LDL, total cholesterol, triglyceride)
( ) Thyroid panel (TSH, T3, T4)
( ) Hemoglobin A1C
( ) Rheumatoid factor (RF)
( ) Hepatitis B serology (HBsAg, anti‑HBs, anti‑HBc, IgM anti‑HBc)
( ) Troponin
( ) Urinalysis
( ) Urine culture
( ) Vitamin D
( ) Vitamin B12
( ) No need for laboratory investigations for this patient

Here you will have the same cases as before but with prices shown. Select the same laboratory investigations before, delete or add laboratory investigations if necessary

9. A 61-year-old male K/C DM, HTN, dyslipidemia. He is currently taking metformin 1000 mg, empagliflozin 10 mg, lisinopril 5 mg, and rosuvastatin 20 mg. He has been brought to your clinic for regular follow-up. The last follow-up was nine months ago. His last labs were unremarkable except for hemoglobin, 11; MCV, 70; HbA1c, 8.1; LDL, 150; and serum creatinine, 90 O/E. The patient is conscious, oriented, and alert. Vitals BP: 132/85; HR: 72; Temp: 37.4; RR: 23; BMI: 29. Local examination was unremarkable. What will you order next for this patient?
( ) Complete blood count with differential (95 SAR)
( ) Coagulation profile (PT, PTT, INR) (192 SAR)
( ) Iron studies (ferritin, transferrin saturation, TIBC) (352 SAR)
( ) Creatinine clearance (100 SAR)
( ) Liver profile (AST, ALT, GGT, albumin) (160 SAR)
( ) Electrolyte panel (CHEM‑7) (200 SAR)
( ) Lipid panel (HDL, LDL, total cholesterol, triglyceride) (160 SAR)
( ) Thyroid panel (TSH, T3, T4) (300 SAR)
( ) Hemoglobin A1C (145 SAR)
( ) Rheumatoid factor (RF) (80 SAR)
( ) Hepatitis B serology (HBsAg, anti‑HBs, anti‑HBc, IgM anti‑HBc) (480 SAR)
( ) Troponin (300 SAR)
( ) Urinalysis (60 SAR)
( ) Urine culture (110 SAR)
( ) Vitamin D (215 SAR)
( ) Vitamin B12 (144 SAR)
( ) No need for laboratory investigations for this patient

10- A 22-year-old woman with a history of asthma presents with a sudden onset of bilateral blindness, denying any trauma. Her mother states, “I can’t believe this is happening. First, her father is dying of cancer, and now this.” On observation, she skillfully avoids obstacles despite her reported blindness. Examination reveals normal bilateral pupillary responses, and there are no signs of bruises or scrapes. A visual evoked potential test was ordered and returned normal. Which laboratory investigations would you order next for this patient?
( ) Complete blood count with differential (95 SAR)
( ) Coagulation profile (PT, PTT, INR) (192 SAR)
( ) Iron studies (ferritin, transferrin saturation, TIBC) (352 SAR)
( ) Creatinine clearance (100 SAR)
( ) Liver profile (AST, ALT, GGT, albumin) (160 SAR)
( ) Electrolyte panel (CHEM‑7) (200 SAR)
( ) Lipid panel (HDL, LDL, total cholesterol, triglyceride) (160 SAR)
( ) Thyroid panel (TSH, T3, T4) (300 SAR)
( ) Hemoglobin A1C (145 SAR)
( ) Rheumatoid factor (RF) (80 SAR)
( ) Hepatitis B serology (HBsAg, anti‑HBs, anti‑HBc, IgM anti‑HBc) (480 SAR)
( ) Troponin (300 SAR)
( ) Urinalysis (60 SAR)
( ) Urine culture (110 SAR)
( ) Vitamin D (215 SAR)
( ) Vitamin B12 (144 SAR)
( ) No need for laboratory investigations for this patient

## References

[1] (2024). About the Ministry of Health Saudi Arabia. https://www.moh.gov.sa/en/Ministry/About/Pages/Budget.aspx.

[2] (2022). National Center for Privatization. https://www.ncp.gov.sa/en/pages/home.aspx.

[3] (2019). Transformation Strategy. https://www.moh.gov.sa/en/Ministry/vro/Documents/Healthcare-Transformation-Strategy.pdf.

[4] Shamashergi M (2021). Practical Implementation in Saudi Arabia’s Healthcare Sector. https://kpmg.com/sa/en/home/insights/2021/02/revenue-cycle-management.html.

[5] (2019). Global Health Saudi. https://www.globalhealthsaudi.com/content/dam/Informa/globalhealthsaudi/downloads/GHE19-KSA-HEALTHCARE-INDUSTRY-OVERVIEW.pdf.

[6] (2022). Private Sector Participation Law. https://www.ncp.gov.sa/en/Pages/Private_Sector_Participation_Law.aspx.

[7] Allan GM, Lexchin J (2008). Physician awareness of diagnostic and nondrug therapeutic costs: a systematic review. Int J Technol Assess Health Care.

[8] Hernu R, Cour M, de la Salle S, Robert D, Argaud L (2015). Cost awareness of physicians in intensive care units: a multicentric national study. Intensive Care Med.

[9] Sá L, Costa-Santos C, Teixeira A (2015). Portuguese family physicians’ awareness of diagnostic and laboratory test costs: a cross-sectional study. PLoS One.

[10] Allan GM, Innes GD (2004). Do family physicians know the costs of medical care? Survey in British Columbia. CFPC.

[11] Long MJ, Cummings KM, Frisof KB (1983). The role of perceived price in physicians' demand for diagnostic tests. Med Care.

[12] Ahmad S, Maqbool A, Srivastava A, Gogoi S, Siddiqui FA, Panwar S (2018). Urine analysis revisited: a review. AIMDR.

